# Tetralogy of Fallot and Complete Atrioventricular Septal Defect with Absent Atrial Component

**DOI:** 10.1016/j.case.2026.04.006

**Published:** 2026-06-02

**Authors:** Matthew Schreier, Helen M. Stanley, Jonathan M. Chen, James Starc, Muhammad Nuri, Lindsay S. Rogers, Laura Mercer-Rosa

**Affiliations:** a Division of Cardiology, Department of Pediatrics, The Children's Hospital of Philadelphia, Philadelphia, Pennsylvania; b Cardiac Center, Nemours Children's Health, Wilmington, Delaware; c Division of Cardiothoracic Surgery, Department of Surgery, The Children's Hospital of Philadelphia, Philadelphia, Pennsylvania

**Keywords:** Echocardiography, Atrioventricular septal defect, Tetralogy of Fallot, Trisomy 21

## Abstract

• AVV anatomy is variable in AVSDs.
• Absent atrial component of the AVSD is associated with a double-orifice common AVV.
• A double-orifice common AVV can be seen in AVSD associated with TOF.
• AVV anatomy can be detailed by 2D and 3D echo to optimize surgical approach.

## Graphical abstract

**Figure:**
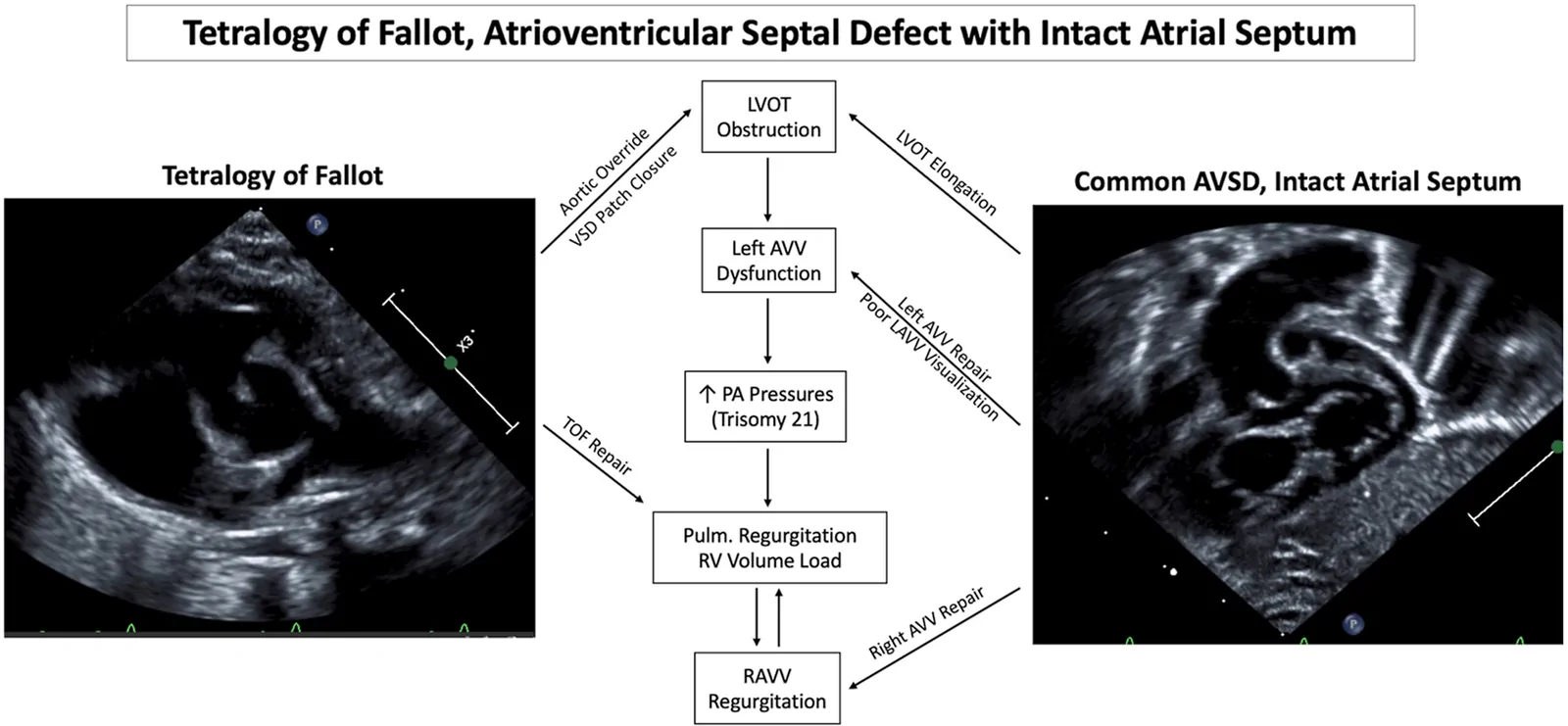

## Introduction

The association of complete atrioventricular septal defect (AVSD) with tetralogy of Fallot (TOF) is a rare but known malformation reported in 0.68% to 1.8% of patients with trisomy 21.^1^^,^^2^ In rare cases of isolated complete AVSD there is absence of the atrial component of the septal defect where the superior and inferior bridging leaflets adhere to the atrial septum, creating 2 separate orifices in the common atrioventricular valve (AVV).^3^^,^^4^ Here we demonstrate the echocardiographic diagnosis and features of a rare presentation of TOF with complete AVSD and absent atrial component of the septal defect with 2 separate AVV orifices in a patient with trisomy 21.

## Case Presentation

A patient born at 37 weeks’ gestation was prenatally diagnosed with TOF with pulmonary atresia by fetal echocardiography and trisomy 21. The patient was born by urgent cesarean section due to prolonged and late decelerations. After initiation of prostaglandins to maintain ductal patency, the patient was transferred to the cardiac intensive care unit, where initial physical examination was notable for a III/VI holosystolic murmur heard throughout the precordium, no hepatosplenomegaly, and normal peripheral pulses without brachiofemoral pulse delay. Examination additionally noted a flattened midface, bitemporal narrowing, small posteriorly rotated ears, a small mouth, excess nuchal skin, and mild hypotonia, consistent with the clinical diagnosis of trisomy 21.

**Figure 1:**
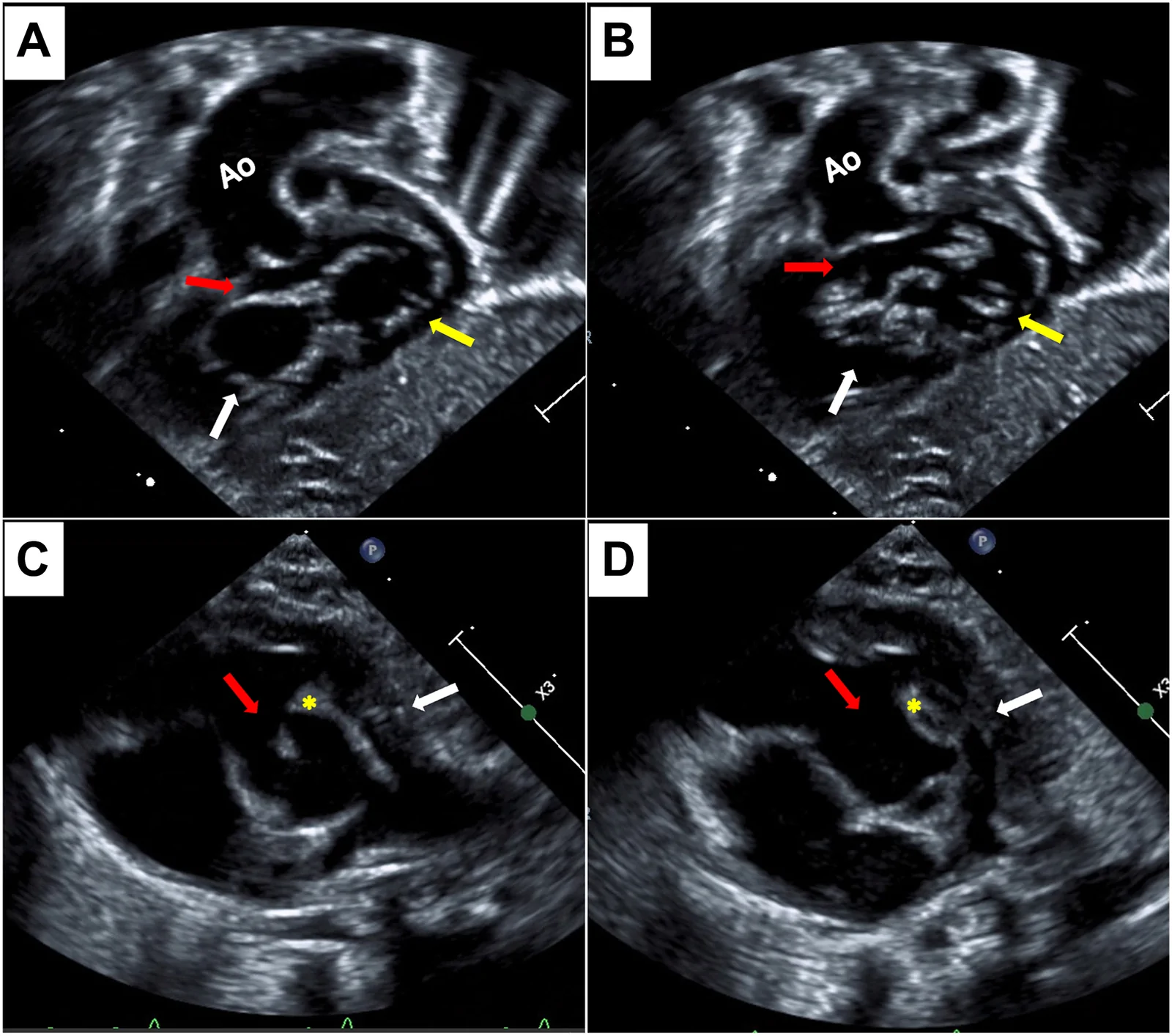
Preoperative 2D TTE, subcostal left anterior oblique **(A, B)** and parasternal short-axis **(C, D)**, diastolic **(A, C)** and systolic **(B, D)** views, demonstrates the aorta (Ao) overriding the ventricular septum, the large VSD *(red arrows)*, and a common AVV divided into 2 separate orifices with the right AVV (*white arrows* in **A, B**) and left AVV (*yellow arrows* in **A, B**) with a trifoliate appearance characteristic of a common AVV, as well as anterior malalignment of the conal septum (∗) with subsequent narrowing of the RVOT and plate-like atresia of the pulmonary valve (*white arrows* in **C, D**) consistent with TOF with pulmonary atresia.

**Figure 2:**
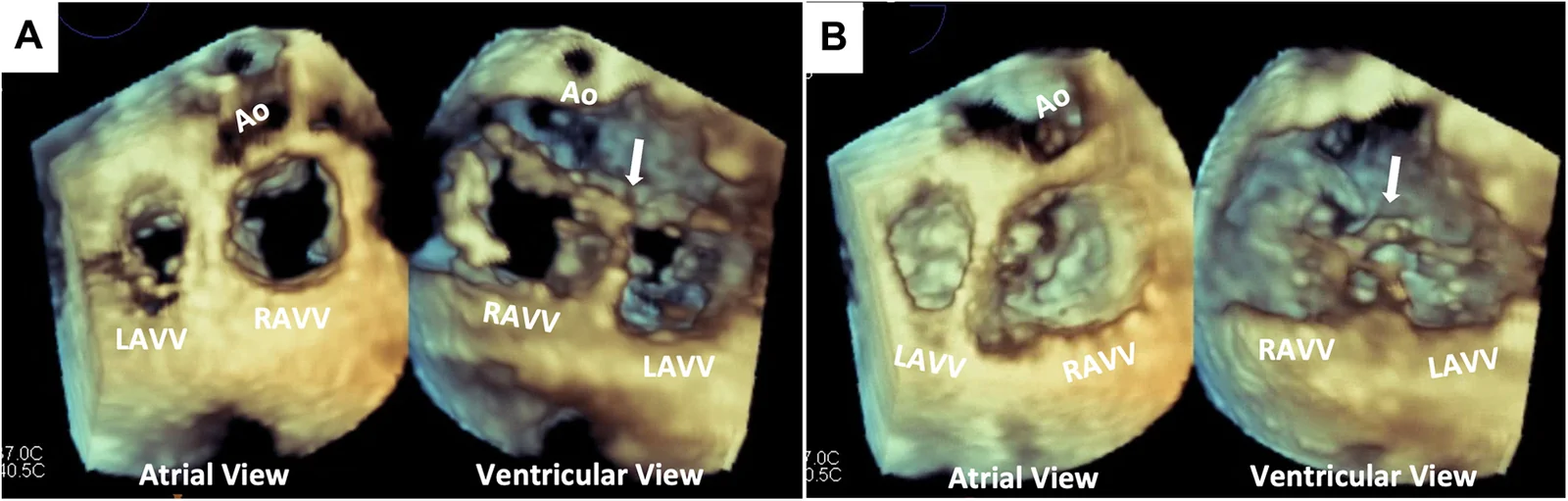
Preoperative 3D TEE, midesophageal short-axis diastolic **(A)** and systolic **(B)** views from the atrial and ventricular perspective, demonstrates the 2 orifices of the common AVV with the trifoliate left AVV (LAVV) and presence of the superior bridging leaflet *(arrow)* seen from the ventricular view consistent with a common AVV. *Ao*, Aorta; *LAVV*, left AVV; *RAVV*, right AVV.

Initial postnatal transthoracic echocardiogram (TTE) confirmed the diagnosis of TOF with a large ventricular septal defect (VSD) characterized by a combination of outlet VSD with anterior malalignment of the conal septum and inlet-type VSD. There was plate-like pulmonary atresia with confluent branch pulmonary arteries. There was a complete AVSD without an identifiable atrial component, with 2 separate orifices to the common AVV and no malposition of the atrial septum (Figure 1, Figure 2, Videos 1-4). Due to concern for a possible collateral artery from the descending aorta in the presence of pulmonary atresia, the patient underwent a computed tomography scan that did not reveal the presence of major aortopulmonary collateral arteries.

Given the ductal-dependent pulmonary blood flow in the context of pulmonary atresia, a right modified Blalock-Taussig-Thomas shunt was performed with surgical end-to-side anastomosis of a 3.5 mm Gore-Tex tube graft to the innominate artery and right pulmonary artery (PA) on day of life 8. The postoperative course was complicated by a respiratory arrest event requiring chest compressions and episodes of hemodynamically significant high-grade heart block, which subsequently self-resolved. The patient underwent stent angioplasty of the proximal Blalock-Taussig-Thomas shunt during this time, narrowed presumably due to the chest compressions. The patient was ultimately discharged home.

Complete repair was performed at 6 months of age. Intraoperatively the diagnosis as described by TTE was confirmed by preoperative transesophageal echocardiogram (TEE) and by the surgeon's visual inspection, and surgical repair was completed with Gore-Tex patch closure of the VSD, placement of an 11 mm pulmonary homograft right ventricular (RV) to PA (RV-PA) conduit, and RV muscle bundle resection. No valvuloplasty was required given the mild left AVV regurgitation noted on preoperative imaging.

**Figure 3:**
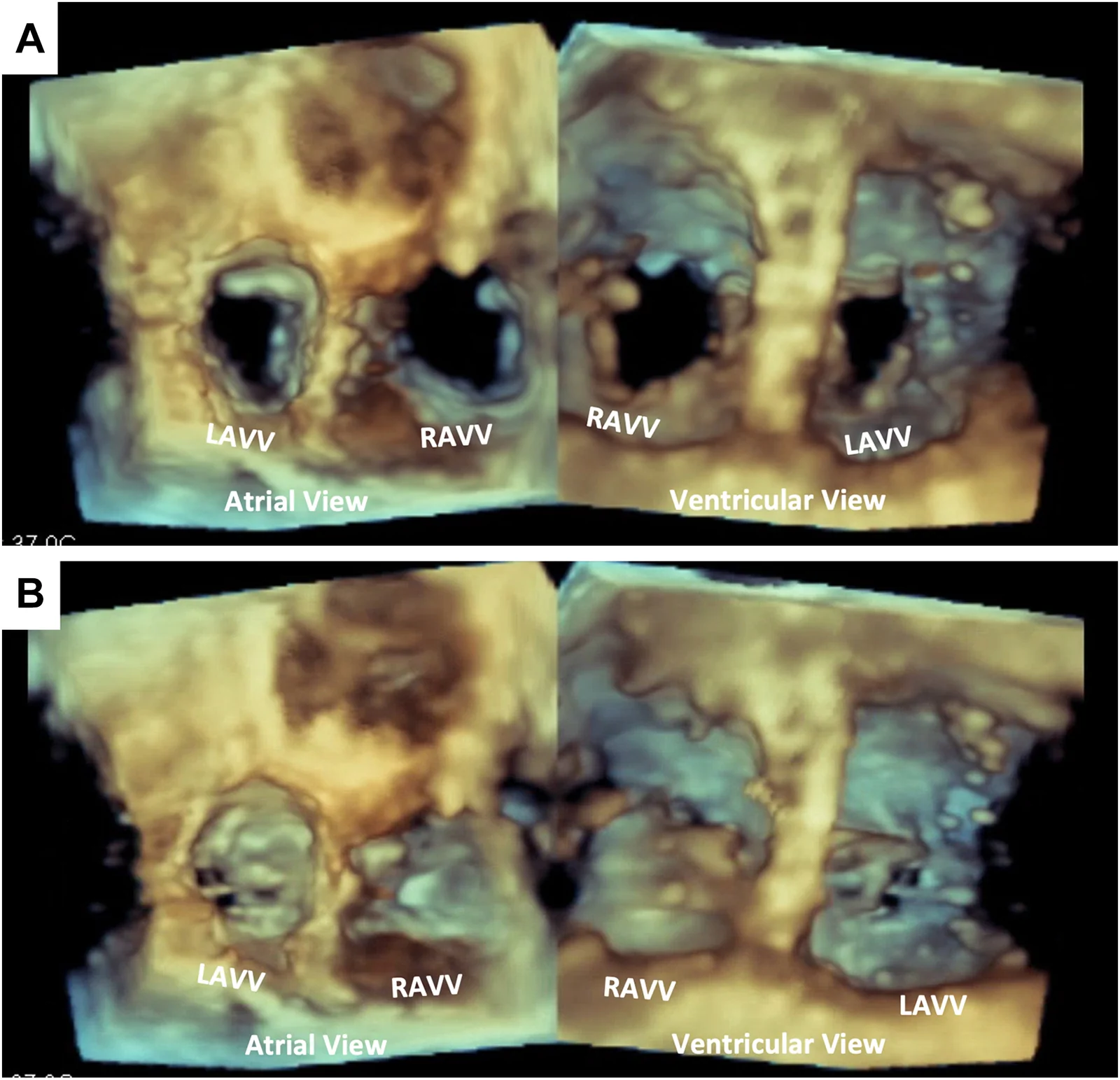
Postoperative 3D TEE, midesophageal diastolic **(A)** and systolic **(B)** views from the atrial and ventricular perspective, demonstrates septated right and left AVVs after VSD patch closure. *LAVV*, Left AVV *RAVV*, right AVV.

Postoperative TEE and subsequent TTE demonstrated trace left and right AVV regurgitation, a mean left AVV inflow gradient of 5.0 mm Hg, mean right AVV inflow gradient of 1.0 mm Hg, and unobstructed outflow tracts with a max velocity of 0.9 m/s (peak gradient 3.0 mm Hg) across the RV-PA conduit and 0.6 m/s (peak gradient 2.0 mm Hg) across the left ventricular outflow tract (LVOT; Figure 3, Video 5).

The postoperative course was complicated by recurrence of high-grade heart block refractory to steroid administration, during which the patient was hemodynamically stable and for which the patient ultimately underwent epicardial single-chamber pacemaker placement and was discharged home. At 3-month follow-up, there had been no cardiac readmissions or reinterventions, and the patient remained asymptomatic with oxygen saturations of 88% to 95%. Pacemaker interrogation demonstrated recovery of normal conduction with 100% ventricular sensing and <0.1% ventricular pacing.

## Discussion

Tetralogy of Fallot, AVSD, and trisomy 21 have a known association; TOF has been noted in 5% to 10% of newborns with AVSD, while AVSD has been noted in 1% to 2% of patients with TOF.^1^^,^^5^, ^6^, ^7^, ^8^ Both of these cardiac malformations have a known association with trisomy 21, with TOF reported in 2.6% to 4.4% of Down syndrome patients and AVSD reported in 12.8% to 17.4% of Down syndrome patients.^2^

The combination of TOF and complete AVSD (TOF-AVSD) is rare but similarly highly associated with trisomy 21, with TOF-AVSD seen in 0.68% to 1.8% of newborns with trisomy 21 and trisomy 21 present in 60% to 85% of patients with TOF-AVSD.^1^^,^^2^^,^^8^ These patients have the common AVV annulus and septal defect inherent to AVSD along with the anterior malalignment of the conal septum, overriding of the aorta over the ventricular septum, and RV outflow tract (RVOT) obstruction characteristic of TOF.^6^^,^^9^ While there is no complete understanding of the mechanism of the relationship between trisomy 21 and these defects, it suggests a common developmental basis of TOF and complete AVSD in trisomy 21 patients.^2^

The combination of TOF and complete AVSD adds diagnostic and surgical complexity to these cases, necessitating thorough preoperative investigation by echocardiography to detail the salient features of the defects. With the presence of significant aortic override over the ventricular component of the septal defect inherent to TOF, there is a risk of causing LVOT obstruction with patch closure of the defect in addition to the LVOT elongation of AVSD, which already puts the LVOT at risk for obstruction.^6^, ^7^, ^8^ Additionally, given the postoperative pulmonary regurgitation that can result after TOF repair, residual right AVV regurgitation can potentially be worsened by the resultant RV volume load.^6^^,^^8^ The residual left AVV regurgitation often seen after AVSD repair in turn can lead to elevated PA pressures, exacerbating pulmonary regurgitation and the consequences of RV volume overload, especially in a trisomy 21 patient population already at risk for higher PA pressures, thus creating a vicious cycle from residual lesions.^6^ The importance of postoperative TEE to detect and quantify the degree of these residual lesions in the operating room to help guide potential surgical reintervention as well as future expectations and planning cannot be overstated.

Outcomes for combined TOF-AVSD surgical repair have significantly improved over the years, with mortality rates reported at 20% to 40% in the 1990s significantly lowered to 0% to 20% in the modern era.^1^^,^^5^ These patients, however, still have high reoperation rates of 20% to 45%, largely associated with residual left AVV regurgitation and residual outflow tract obstruction.^1^^,^^3^^,^^5^^,^^8^ Freedom from RVOT reintervention, a known concern in TOF, has been reported at 85.9% at 10 years and 79.2% at 20 years in TOF-AVSD, similar to large-size reports of freedom from pulmonary valve replacement at 95.5% and 80.1% and other RVOT intervention of 97% and 95.9% at 10 and 20 years, respectively, in isolated TOF.^1^^,^^10^

Additionally, this compilation of lesions carries a risk of conduction abnormalities. Complete AVSD characteristically has inferior and posterior displacement of the conduction system, and it has been hypothesized that this is similar in cases with an intact atrial component.^3^^,^^11^ This suboptimal conduction system in AVSD is believed to predispose patients to heart block and is known to be even more vulnerable to injury during surgery, with a 4% incidence of heart block in patients both with and without trisomy 21.^11^ This has also been variably shown in TOF-AVSD patients with heart block rates of 0% to 5%.^5^^,^^8^^,^^12^ Notably, heart block in AVSD is associated with higher incidence of mortality after surgery.^11^

Notable to this case is the absence of the atrial component of the AVSD with the resultant 2 orifices of the common AVV noted on the initial postnatal echocardiogram. The atrial component of the septal defect is absent in very rare cases of isolated complete AVSD, but this has not been reported in major institutional experiences of TOF-AVSD.^1^^,^^3^, ^4^, ^5^, ^6^, ^7^, ^8^^,^^13^ In isolated AVSD, absence of the atrial component of the septal defect appears to be more common in patients with trisomy 21; however, the 1 reported case of TOF-AVSD with absence of the atrial component of the septal defect was noted in a patient without trisomy 21.^3^^,^^9^

In cases of complete AVSD where the atrial component is absent, the superior and inferior bridging leaflets of the common AVV leaflets have attachments to the atrial septum, creating 2 separate orifices of the common AVV. The absence of distinct fibrous rings around each AVV and the trifoliate morphology of the left AVV confirm this as a common AVV and not 2 separate mitral and tricuspid valves.^3^^,^^4^ This in turn differs from the equally rare variation of double-orifice left and right AVV, in which there is a double orifice on either the left ventricular or RV component of the common AVV (thus 3 orifices are seen), rather than 2 distinct orifices dividing the common AVV along the point of arrested septation as described here.^14^^,^^15^

Absence of the atrial component of the AVSD further increases the challenges of diagnosis and surgical correction. As seen in this case, the fetal echocardiogram made the diagnosis of TOF but not AVSD, with the intact atrial septum and 2 orifices of the AVV making it challenging to delineate this as a common AVV prenatally. This necessitates close attention to the AVV morphology on postnatal TTE to allow appropriate preoperative planning.

Surgically, the lack of a transseptal window for exposure of the left AVV caused by absence of the atrial component of the septal defect adds to the technical difficulty of repairing this valve.^3^ Additionally, potential malposition of the atrial septum, with leftward malposition being more commonly reported, may lead to a small left AVV, which can further impede the ability for cleft closure without causing left AVV stenosis. This also predisposes these patients to left AVV regurgitation, making it crucial to detail the atrial septal anatomy on preoperative echocardiography and to delineate the presence of any malposition of the atrial septum.^3^^,^^4^ Not only does this put these patients at risk for need of reoperation, but when coincident with TOF it could increase the risk for elevated left atrial and PA pressures with resultant impact on pulmonary regurgitation and RV volume load as described above.^3^^,^^6^

Here we report the echocardiographic diagnosis of a rare case of TOF and complete AVSD with absent atrial component and a common AV valve with 2 separate orifices in a patient with trisomy 21. While exceedingly rare, the additional challenges presented by this configuration require close echocardiographic attention to valve and septal anatomy when planning surgical intervention for these already complex patients to ensure optimal outcomes as well as careful echocardiographic delineation of residual lesions postoperatively to guide future management.

## Conclusion

The particular combination of TOF and a complete AVSD with absence of the atrial component of the septal defect in this patient with trisomy 21 is exceedingly rare. This unique cardiac defect adds complexity to the diagnosis and surgical repair and the risk for reinterventions. Careful preoperative echocardiographic evaluation of the AVSD morphology using two- and three-dimensional (2D and 3D) imaging is essential to optimize the surgical approach. Careful postoperative imaging is crucial to limit and guide treatment for the myriad of complications exacerbated by residual lesions in these patients.

## Ethics Statement

The authors declare that the work described has been carried out in accordance with The Code of Ethics of the World Medical Association (Declaration of Helsinki) for experiments involving humans.

## Consent Statement

The authors declare that since this was a non-interventional, retrospective, observational study utilizing de-identified data, informed consent was not required from the patient under an IRB exemption status.

## Disclosure Statement

The authors report no conflict of interest.

## Supplementary Data

Video 1 Preoperative 2D TTE, subcostal left anterior oblique view, demonstrates the aorta overriding the ventricular septum, a large VSD, and a common AVV divided into 2 separate orifices, with the right AVV and left AVV with a trifoliate appearance characteristic of a common AVV.

Video 2 Preoperative 2D TTE, parasternal short-axis view, demonstrates anterior malalignment of the conal septum with subsequent narrowing of the RVOT and plate-like atresia of the pulmonary valve consistent with TOF with pulmonary atresia.

Video 3 Preoperative 2D TTE, parasternal long-axis sweep, demonstrates the aortic override of the ventricular septum, fibrous continuity of the aortic valve with the left AVV, a VSD (combination inlet and outlet with anterior malalignment of the conal septum), and presence of a superior bridging leaflet consistent with an AVSD.

Video 4 Preoperative 3D TEE, midesophageal short-axis views from the atrial *(left)* and ventricular *(right)* perspective, demonstrates the 2 orifices of the common AVV with the trifoliate left AVV, no atrial component of the canal defect with 2 separate orifices of the common AVV, and presence of the superior bridging leaflet seen from the ventricular view consistent with a common AVV.

Video 5 Postoperative 3D TEE, midesophageal views from atrial *(left)* and ventricular *(right)* perspective, demonstrates septated right and left AVVs after VSD patch closure.
